# Rescue from tau-induced neuronal dysfunction produces insoluble tau oligomers

**DOI:** 10.1038/srep17191

**Published:** 2015-11-26

**Authors:** Catherine M. Cowan, Shmma Quraishe, Sarah Hands, Megan Sealey, Sumeet Mahajan, Douglas W. Allan, Amritpal Mudher

**Affiliations:** 1 Centre for Biological Sciences, University of Southampton, Southampton, SO17 1BJ, UK; 2 Institute of Life Sciences and Department of Chemistry, University of Southampton, Southampton SO17 1BJ, UK; 3 Department of Cellular and Physiological Sciences, Life Science Institute, University of British Columbia, Vancouver, V6T 1Z3, Canada

## Abstract

Aggregation of highly phosphorylated tau is a hallmark of Alzheimer’s disease and other tauopathies. Nevertheless, animal models demonstrate that tau-mediated dysfunction/toxicity may not require large tau aggregates but instead may be caused by soluble hyper-phosphorylated tau or by small tau oligomers. Challenging this widely held view, we use multiple techniques to show that insoluble tau oligomers form in conditions where tau-mediated dysfunction is rescued *in vivo*. This shows that tau oligomers are not necessarily always toxic. Furthermore, their formation correlates with increased tau levels, caused intriguingly, by either pharmacological or genetic inhibition of tau kinase glycogen-synthase-kinase-3beta (GSK-3β). Moreover, contrary to common belief, these tau oligomers were neither highly phosphorylated, and nor did they contain beta-pleated sheet structure. This may explain their lack of toxicity. Our study makes the novel observation that tau also forms non-toxic insoluble oligomers *in vivo* in addition to toxic oligomers, which have been reported by others. Whether these are inert or actively protective remains to be established. Nevertheless, this has wide implications for emerging therapeutic strategies such as those that target dissolution of tau oligomers as they may be ineffective or even counterproductive unless they act on the relevant toxic oligomeric tau species.

All tauopathies, including Alzheimer’s disease (AD), are characterized by the accumulation of insoluble, hyper-phosphorylated aggregates of the microtubule-associated protein tau. Both tau aggregation and hyper-phosphorylation are implicated in tau-mediated dysfunction and toxicity^1^. Hence, research focuses on developing therapies to inhibit aggregation or hyper-phosphorylation^1^,^2^.

Tau can be phosphorylated at a large number of sites, and many of these sites are abnormally hyper-phosphorylated in AD^3^. Various serine-threonine kinases have been implicated in tau hyper-phosphorylation including glycogen synthase kinase 3β (GSK-3β)^4^,^5^,^6^. We have previously shown that soluble tau that is highly phosphorylated at GSK-3β sites causes neuronal dysfunction by destabilizing cytoskeletal integrity, impairing axonal transport and disrupting synaptic function^7^,^8^,^9^. Others have similarly reported phospho-tau mediated neuronal dysfunction in various animal models of tauopathy^10^,^11^,^12^. As well as causing dysfunction, soluble hyper-phosphorylated tau has been shown to be directly toxic triggering degeneration and neuronal loss^13^,^14^,^15^,^16^. Some studies have also reported that hypo-phosphorylation of tau may also be toxic^17^, perhaps due to dysregulation of microtubules, which will have the same effect as hyper-phosphorylated tau by impacting axonal transport and synaptic function^18^. Overall, the causal pathogenic role played by soluble hyper-phosphorylated tau is well documented by many studies and thus largely undisputed.

In contrast, the case for tau aggregates as a primary toxic species is less clear. Indeed the toxicity of aggregates has been challenged for other aggregating proteins in other proteinopathies as well^19^,^20^,^21^,^22^. In AD brains and animal models, a wide range of tau aggregates of varying size, morphology and solubility have been identified. These range from soluble dimers and small oligomers^23^, to larger insoluble granular tau oligomers (GTOs) of approximately 40 tau units^24^ that are assumed to be precursors of the protofibrils which ultimately form neurofibrillary tangles. Though tangle pathology correlates with cognitive decline in AD, results from animal models have raised questions about their toxicity^25^,^26^,^27^. For example in inducible tau transgenic mice, both memory deficits^28^ and neuronal loss^29^ are rescued by switching off tau transgene expression and yet tangle pathology persists. Following such findings, the search for the toxic tau aggregates deviated from tangles to their precursors, the tau oligomers. Tau oligomers have been described in early stages in AD brains^30^,^31^ and in transgenic models of tauopathy^32^,^33^. Several studies imply that they mediate tau toxicity in tauopathies^34^. For example tau oligomerisation closely correlates with memory loss in a transgenic model of tauopathy^32^ and stereotaxic injection of recombinant tau oligomers but not monomers or fibrils impairs learning and memory in wild-type mice^35^. In the latter study, the tau oligomers also caused significant neuronal death around the injection site. Thus oligomeric tau species are now seriously being considered as targets of tau-based therapeutic strategies^34^,^36^.

Though the ever-increasing studies on tau oligomers clearly describe a variety of oligomers that differ in size (and number of tau protein constituents), shape and solubility, these differences are rarely acknowledged or discussed. Consequently their contribution to the pathogenic potential of oligomeric tau species is not fully appreciated^26^. Instead tau oligomers are generally considered to be a toxic species of tau comprised of highly phosphorylated and aggregated tau. The results we describe in this paper challenge this view and thus highlight the need for scientists of future studies to more clearly characterize and describe the oligomeric tau species they are working on. We show that insoluble tau oligomers, comprising of non-phosphorylated tau can form *in vivo* in situations where tau-mediated neuronal dysfunction is rescued. Thus tau oligomers are not necessarily made up of hyper-phosphorylated tau and they are not necessarily associated with tau toxicity.

## Results

### Rescue of tau-induced phenotype led to formation of structures resembling tau oligomers

**Figure 1:**
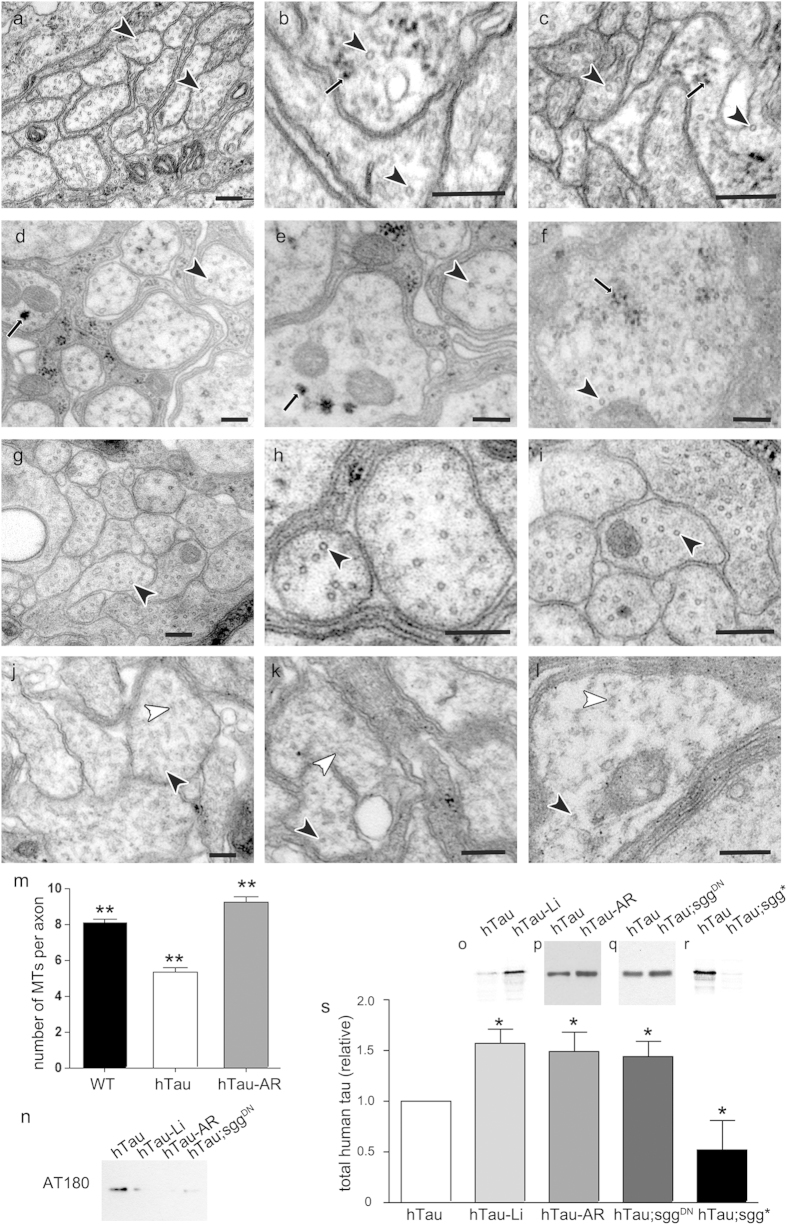
GSK-3β inhibition rescued microtubule number in hTau^0N3R^ *Drosophila*, but increased total hTau protein and caused formation of electron-dense granules a-l) Electron micrographs of transverse sections of peripheral nerves in L3 *Drosophila* (scale bar 200 nm). In hTau-expressing *(elav*^*C155-*^*Gal4*/*Y; UAS-hTau*^*0N3R*^/+) animals treated with either 20 mM LiCl (hTau-Li, a–c) or with 20 μM AR-A01448 (hTau-AR, d-f), some axons exhibited small electron-dense globular structures of approximately 20–50 nm in size (black arrows). These structures were extremely rare in control larvae expressing *elav*^*C155*^*-Gal4* driver alone (WT, **g**–**i**) or untreated hTau^0N3R^-expressing neurons (**j**–**l**). In WT larvae the axon profiles showed numerous regularly-spaced, correctly-aligned transverse microtubule profiles (black arrowheads in g-i; 8.1 ± 0.2/axon profile). As we have previously shown^29^, in hTau^0N3R^-expressing axons the microtubules were dramatically disrupted, with fewer correctly-aligned transverse microtubule profiles (black arrowheads in j-l; 5.3 ± 0.3/axon profile), and evidence of disorganised microtubules in the same axon profiles (white arrowheads in **j**–**l**). Indeed, approximately 30% of hTau^0N3R^-expressing axons displayed no visible microtubule profiles (Figure S1). In hTau^0N3R^-expressing larvae fed with Li (**a**–**c**) or AR (**d**–**f**), there were significantly more correctly-aligned transverse microtubule profiles (black arrowheads in a-l; 9.2 ± 0.3/axon) and fewer misaligned microtubules. Microtubule numbers per axon are quantified in m (**p < 0.01, unpaired Students t test). Representative Western blots of hTau^0N3R^-expressing fly head lysates showed that tau phosphorylation was decreased (at T231/S235 detected by AT180) whilst total tau levels were increased by 40–60% (**o**–**r**) by 20 mM lithium treatment (hTau-Li, o), 20 μM AR-A01448 treatment (hTau*-*AR, p), co-expression of dominant negative *shaggy* (hTau*;sgg*^*DN*^, q) {*elav*^*C155*^*-Gal4*/*Y; UAS- hTau*^*0N3R*^/* *+* ; UAS-sgg*^*DN*^/* *+* *}. Conversely, total tau levels were decreased by approximately 50% by co-expression of constitutively active shaggy (hTau;sgg*, r) *{elav*^*C155*^*-Gal4*/*Y; UAS- hTau*^*0N3R*^/* *+ ; UAS-sgg*/* *+* *}. This is quantified in s (error bars are standard error of mean; *p < 0.05 by Students t-test).

We have previously shown that reduction of GSK3β-mediated tau phosphorylation (using LiCl or a more specific GSK-3β inhibitor, AR-A01448) rescues phenotypes induced by human tau (hTau^0N3R^) in *Drosophila*. These phenotypes include locomotor impairment and disrupted axonal transport^7^,^8^,^9^,^37^,^38^. While examining the ultrastructure of hTau^0N3R^-expressing neurons in these animals, we made an unexpected observation: treatment with either drug led to the formation of 20–50 nm electron-dense granules in axonal EM sections (Fig. 1, arrows in 1a-f and quantified in Supplementary Fig. 1). These structures bear a striking resemblance to granular tau oligomers (GTOs) first described by the Takashima group in AD brains^30^. Subsequent *in vitro* characterization by their group proved that AD brain GTOs weigh 1800 kDa and contain on average 40 molecules of tau^24^,^39^. Here, we tested the hypothesis that the electron-dense granules we observed in hTau-expressing *Drosophila* after GSK-3β inhibition are indeed GTO-like structures.

We first ascertained whether these GTO-like structures were associated with rescue of phospho-tau-mediated phenotypes. Therefore we examined whether like LiCl^8^,^9^, AR-A01448, which also produces GTOs and rescues axonal transport and locomotion (Supplementary Fig. 2), similarly restores cytoskeletal integrity. Figure 1 and Supplementary Fig. 3 show that microtubules in AR-A01448-treated hTau^0N3R^ larvae (black arrowheads in Fig. 1d–f) were indistinguishable from control in number and integrity (black arrowheads in Fig. 1g–i). In marked contrast, axons of untreated hTau^0N3R^ animals displayed reduced numbers and misaligned microtubules, as previously reported^8^,^9^ (white arrowheads in Fig. 1j–l. Quantified in Fig. 1m and Supplementary Fig. 3). This demonstrates that functional rescue by GSK-3β inhibition (both LiCl and AR-A01448) is sufficient to restore microtubule integrity thus rescuing tau-induced neuronal dysfunction. Remarkably, rescuing tau-induced phenotypes by this mechanism also resulted in formation of GTO-like structures.

### Inhibition of tau phosphorylation led to increased tau levels

Since LiCl and AR-A01448 treatments produce GTO-like structures, which we hypothesize contain tau, we assessed human tau levels in animals treated with these drugs. We observed an intriguing increase of total hTau in response to GSK-3β inhibition. Treatment with either LiCl (Fig. 1o) or AR-A01448 (Fig. 1p) not only predictably reduced tau phosphorylation (Fig. 1n), but also significantly increased hTau levels by 50% (Fig. 1s). Genetic manipulation of GSK-3β confirmed this. Co-expression of hTau^0N3R^ with a dominant-negative allele of *shaggy* (*sgg*^*DN*^), the *Drosophila* homolog of GSK-3β, similarly reduced tau phosphorylation (Fig. 1n) and significantly increased hTau levels (Fig. 1q,s; for representative loading controls of blots in Fig. 1o–n see Supplementary Fig. 4). Conversely, expression of constitutively-active *shaggy* (sgg*; which increases tau phosphorylation and exacerbates the behavioural phenotype^8^,^9^) reduced hTau levels (Fig. 1r,s). This shows that GSK-3β inhibition increases hTau levels, whilst GSK-3β activation decreases it. This effect is specific to GSK-3β because treatment with a microtubule-stabilising peptide davunetide/NAP rescues tau phenotypes but does not alter tau levels^38^. Also, inhibition of GSK-3β does not non-specifically increase levels of other transgenes (Supplementary Fig. 5) showing that this is a human tau-specific effect. Our result is counter-intuitive, since many individuals with tauopathies exhibit elevated tau levels^40^. In contrast, we observed elevated tau levels in conditions where tau phenotypes were rescued, and lower levels where the phenotypes were exacerbated. We propose that high tau levels are not detrimental when low levels of tau phosphorylation are maintained. Elevated tau levels could conceivably cause GTO formation in our model, as aggregation of tau has been shown to be critically dependent upon tau concentration^41^.

### Multiple techniques confirmed that GTO-like structures were authentic tau oligomers

We tested our hypothesis that the GTO-like structures formed following GSK-3β inhibition are indeed composed of insoluble tau using several approaches including biochemical assays, immuno-gold EM and Atomic Force Microscopy (AFM).

**Figure 2:**
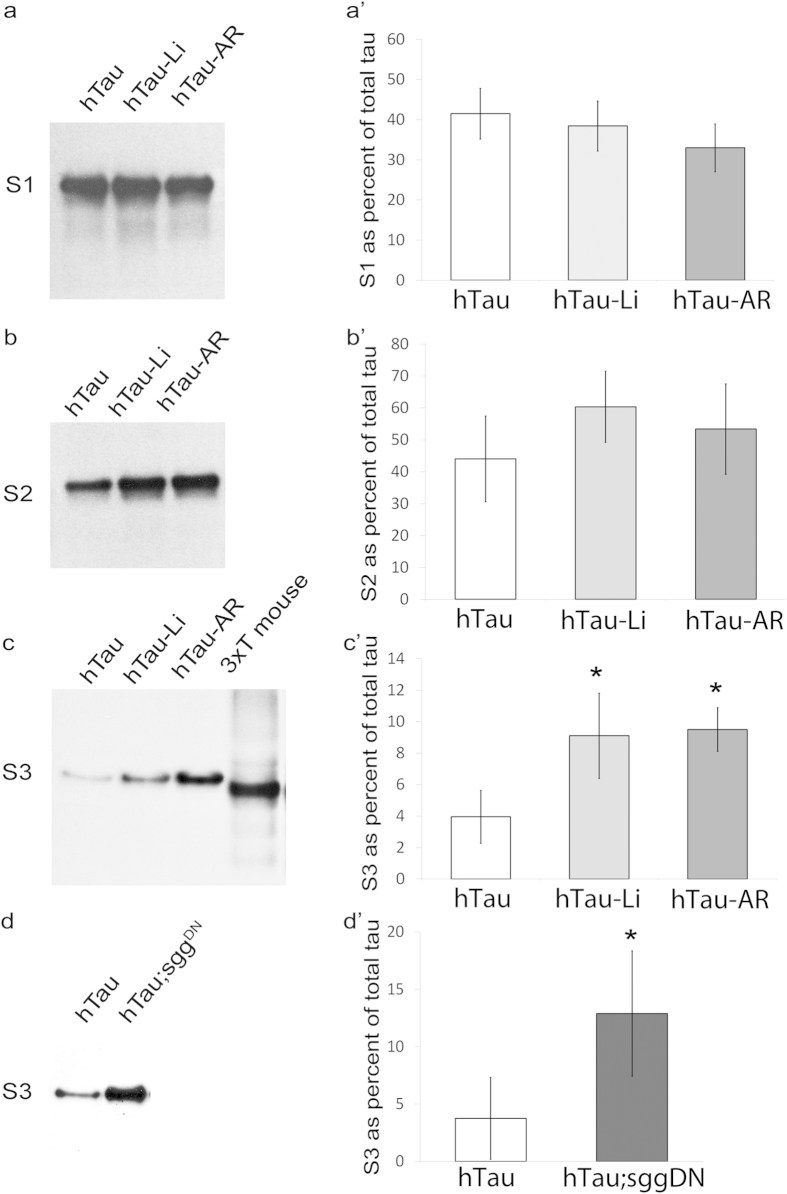
The amount of insoluble tau detected from hTau^0N3R^-expressing *Drosophila* is increased dramatically after treatment with GSK-3β inhibitors. Western blots of aqueous-soluble fraction (S1), detergent-soluble fraction (S2) and insoluble fraction (S3) probed for anti-hTau. Samples in lanes 1–3 are from heads of hTau^0N3R^ flies (hTau – {*elav*^*C155-*^*Gal4*/*Y ; UAS-hTau*^*0N3R*^/* *+* *}), hTau^0N3R^ flies treated with 20 mM lithium (hTau-Li), hTau^0N3R^ flies treated with 20 μM AR-A01448 (hTau-AR), and flies co-expressing hTau^0N3R^ with dominant negative *shaggy* (hTau;*sgg*^DN^ {*elav*^*C155*^*-Gal4*/*Y; UAS- hTau*^*0N3R*^/+; *UAS-sgg*^*DN*^/* *+* *}). The fourth lane in the third panel (labelled “3xT mouse”) is a 10-fold dilution of sample from triple-transgenic mouse, used as a positive control for insoluble tau. Bar charts in a’–d’ are quantifications of blots in a – d presented as a percentage of total tau {S3/(S1 + S2 + S3)} in each genotype. Treatment with Li or AR-AR01448 did not alter the amount of tau detected in either the S1 (**a**,**a**’) or S2 fractions (**b**,**b**’). However treatment with Li, AR-AR01448 (**c**,**c**’) or co-expression of dominant negative *shaggy* (hTau;*sgg*^DN^) (**d,d’**) significantly increased the amount of tau detected in the insoluble S3 fraction. (error bars are standard error of mean and n = 5 for each genotype/treatment; *p < 0.05 by Students t-test).

First, we hypothesized that if GTO-like structures were forming then we would identify them using commonly used biochemical assays used to detect insoluble tau oligomers. Indeed the presence of tau immuno-positive material within the stacking gel of brain lysates from drug treated hTau^0N3R^ flies suggested that insoluble tau species were present but were unable to enter the SDS-resolving gel (Supplementary Fig. 6). To explore this further, we fractionated brain lysates into aqueous-soluble (S1), detergent-soluble (S2) and detergent-insoluble (S3) fractions using established protocols for identifying insoluble tau oligomers^24^,^39^ (Supplementary Fig. 7 shows that this protocol detects insoluble tau). In all hTau^0N3R^ flies, whether drug treated or not, the majority of hTau was in the S1 aqueous-soluble (Fig. 2a) or S2 detergent-soluble fractions (Fig. 2b). The fraction of tau (as a percentage of total tau) found in either S1 or S2 was the same for all hTau^0N3R^ flies, whether drug treated or not (Fig. 2a’). In contrast, the amount of tau found in the insoluble S3 fraction changed significantly following drug treatment. Whereas only a very small amount of tau was detected in the insoluble S3 fraction in untreated flies (Fig. 2c htau lane and Fig. 2c’ white bar), this increased dramatically, almost by three-fold, after LiCl or AR-A01448 treatment (Fig. 2c htau-Li and htau-AR lanes and Fig 2c’ light gray and dark gray bars). To confirm that the protocol used to generate the S3 fraction was indeed enriching for insoluble aggregated tau, it was used to fractionate tau from transgenic mice where such tau is found in abundance. As expected, insoluble tau was detected with our protocol from tau transgenic brain homogenate (Fig. 2c 3xT lane). To verify that the increase in insoluble tau was caused by GSK-3β inhibition and not any other drug action, we assessed tau solubility in hTau^0N3R^;Sgg^DN^ flies. Co-expression of Sgg^DN^ led to a significant increase in insoluble tau, proving that this effect was mediated by GSK-3β inhibition (Fig. 2d and d’). These results were corroborated using a second anti-tau antibody, which confirmed that the material picked up in the S3 fraction of drug treated hTau^0N3R^ flies was indeed insoluble tau (Supplementary Fig. 6b). As mentioned above, this effect is specific to GSK-3β because davunetide/NAP treatment neither produced GTO-like structures^38^ nor increased insoluble tau levels (Supplementary Fig. 6c). This data shows that pharmacological or genetic reduction of GSK-3β-mediated tau phosphorylation leads to increased tau levels and formation of structures containing insoluble tau.

**Figure 3:**
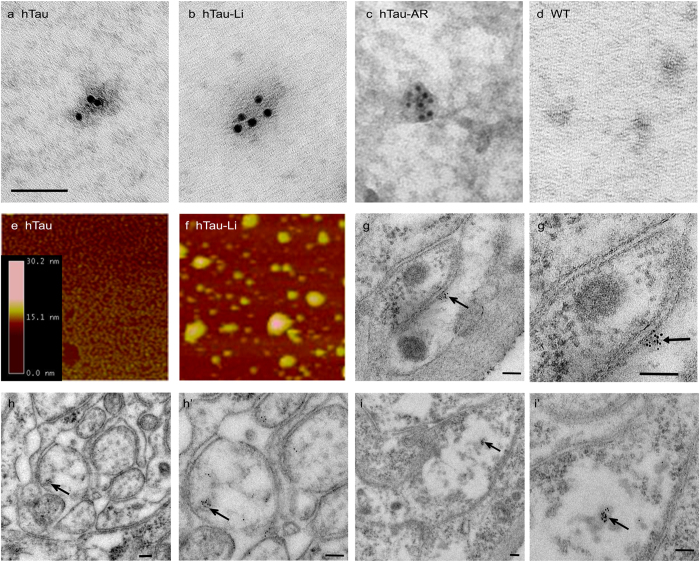
Insoluble tau oligomers can be purified from hTau^0N3R^-expressing *Drosophila* and were increased dramatically after treatment with GSK-3β inhibitors. TEM of immuno-gold labelling for anti-hTau of S3 insoluble fractions show granular tau oligomers comprised of hTau in all conditions expressing hTau {*elav*^*C155-*^*Gal4*/*Y; UAS-hTau*^*0N3R*^/* *+* *}: (**a**) hTau, (**b**) hTau-Li, (**c**) hTau-AR. No such structures were detected in controls: d) wild-type. (See also Supplementary Fig. 8, for additional controls of no sample labelled with anti h-Tau; and hTau labelled for an irrelevant rabbit polyclonal antibody, anti-v-glut). Scale bar in a (applicable to **a**–**f**) = 100 nm. (**g**–**h**); hundreds of such structures were observed in preparations from 18 pooled flies) Atomic Force Microscopy of material immuno-precipiated from fly head lysates using anti-hTau antibody shows the appearance of numerous granular tau oligomers present after LiCl treatment (**f**) but only very sparse in untreated hTau^0N3R^ flies (**e**). Oligomer sizes were determined by cross-sectional height analysis of individual oligomers. The heights of the oligomers ranged between 15 and 30 nm, with a mean height of 17.07 nm (SD = 8.86). The widths of the majority of oligomers are between 20 and 40 nm and the average width was calculated to be 20.6 nm (SD = 11.4). A minority of oligomers have a larger width than 30 nm but the height of the oligomers was consistently 30 nm or below. Scale bar in i = 1 μm. (**g**–**i**)’) Immuno-gold labeling for hTau *in situ* in sections of peripheral nerves from hTau-Li flies demonstrates labeled granular tau oligomers (arrows) within axons. Examples are given at lower magnifications (**g**–**i**) in which axonal profiles are clearer, and at higher magnifications (**g**’–**i**’) in which GTOs can be seen more clearly. Scale bars = 100 nm.

To further confirm that these structures contain tau, we performed immuno-gold EM on insoluble fractions. In all hTau^0N3R^-expressing conditions we detected 20–50 nm granular oligomeric structures decorated with anti-hTau antibody (Fig. 3a–c). No labeling was evident in non-transgenic or antibody controls (Fig. 3d and Supplementary Fig. 8). In line with the results from the EM (Fig. 1) and biochemical analyses (Fig. 2), many more such structures were evident in the drug treated hTau^0N3R^ animals than in untreated controls. These data strongly imply that insoluble tau granules, GTO-like structures, are forming in drug-treated hTau^0N3R^ flies.

To corroborate this and to accurately measure the size of the granular structures, we subjected hTau immuno-precipitated from fly head lysates to atomic force microscopy (AFM), a procedure routinely used to visualise tau oligomers^24^. Abundant granular spherical structures were observed in samples from LiCl-treated hTau^0N3R^ brains (Fig. 3f), but were extremely sparse in untreated hTau flies (Fig. 3e). The dimensions of these structures were strikingly similar to GTOs identified from AD brains^24^,^30^, varying in width from 5–50 nm, with an average width and height of 20 nm. This data strongly indicates that inhibition of GSK-3β in hTau^0N3R^ flies produces insoluble GTO-like structures similar to those found in AD brain.

To confirm that these GTO-like structures isolated biochemically represent the same structures as the electron-dense granules observed *in situ*, we performed *in situ* immuno-gold labeling for hTau on sections of peripheral nerve. In conditions in which electron-dense granules were observed, such as Li-treated hTau flies, we found granules decorated with gold particles within axons (arrows in Fig. 3g–i).

Collectively, the results from all the above assays prove our hypothesis by demonstrating that inhibition of GSK-3β produces insoluble oligomers showing GTO-like structure.

### hTau in GTO-like structures was not phosphorylated

**Figure 4:**
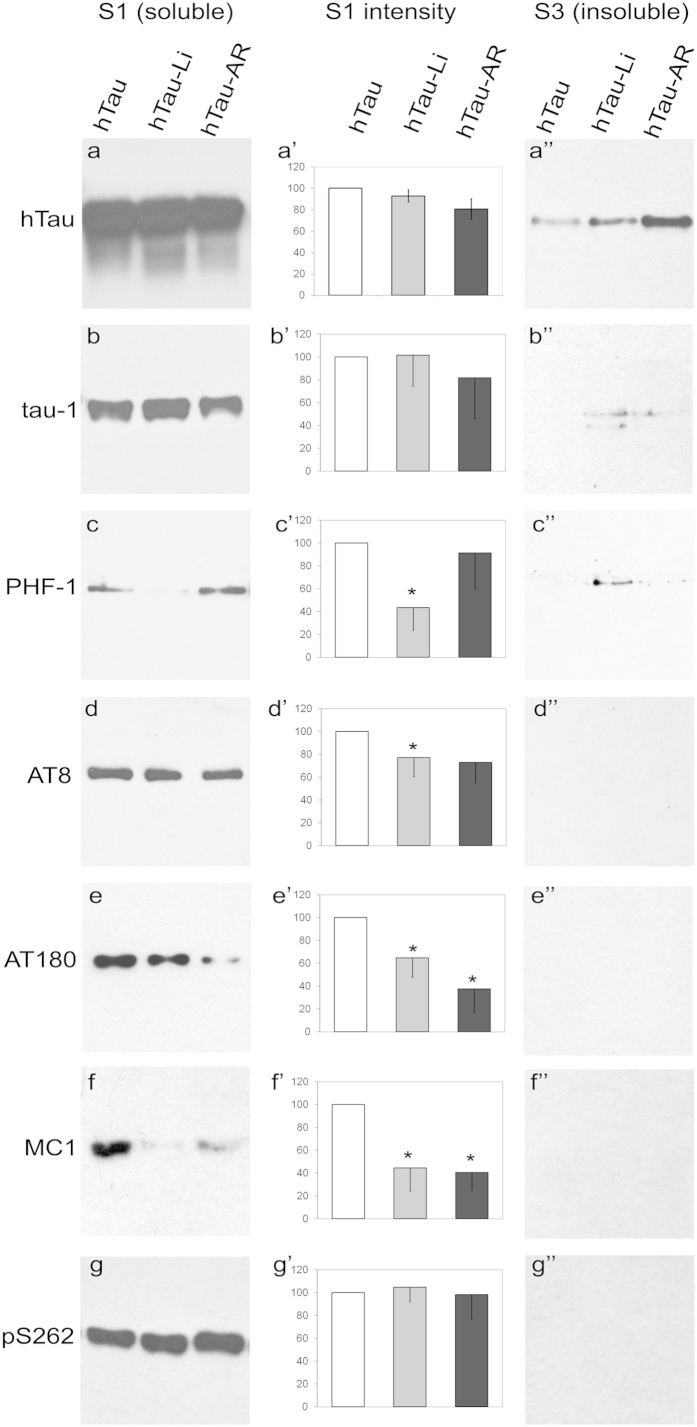
Tau within GTO-like structures is largely unphosphorylated Western blots of the soluble fraction (S1) and the insoluble fraction (S3) from hTau {*elav*^*C155*^*-Gal4*/Y; UAS- hTau^0N3R^/ + }, hTau-Li and hTau – AR-A01448 treated fly brain lysates probed with an antibody that detects total tau (a and a”) and those that detect various phospho-tau epitopes (b-g and b”-g”). Though there is a significant amount of tau in the insoluble fraction of drug treated brain lysates (**a**”), it is largely unphosphorylated at many sites (**b**”–**g**”). Signal at these sites in the soluble fractions (**b**–**g** and **b**’–**g**’) provides a positive control for the antibodies, and shows that treatment with GSK-3β inhibitors LiCl and AR-A01448 reduced phosphorylation at many of these sites: quantified in graphs (**b**’–**g**’). Graphs show the average of 6 independent experiments; error bars are SEM; * p < 0.05. Blots were probed with (**a**) dako polyclonal anti-tau (total tau), (**b**) anti-tau-1 (unphosphorylated at 192–204), (**c**) anti-PHF-1 (pS396, pS404), (**d**) anti-AT8 (pS202, pT205), (**e**) anti-AT180 (pT231, pS235), (**f**) anti-MC1 (it is curious that this supposedly conformation specific anti-body picks up its epitope after SDS-PAGE denaturation; it is likely that the epitope recognized here is largely denatured but nonetheless is disease predictive since others, like us have also shown similar WB immunoreactivity in another *Drosophila* model of tauopathy^10^), (**g**) anti-pS262.

It is often presumed that tau aggregates including oligomers are composed of phosphorylated tau. However we show here that decreased phosphorylation leads to formation of insoluble tau oligomers. Therefore we determined whether these insoluble oligomers are in fact phosphorylated by assessing the phosphorylation status of hTau in soluble and insoluble fractions from fly heads. We found that tau in the soluble fraction was highly phosphorylated in hTau^0N3R^ flies, at many of the sites hyper-phosphorylated in AD (htau lanes in Fig. 4b–g). As expected, GSK-3β inhibition significantly reduced tau phosphorylation at several of these sites except at the ser262 site (htau-Li and htau-AR lanes in Fig. 4b–g and Fig. 4b’–g’ where reduction in phosphorylation in drug-treated hTau^0N3R^ flies is quantified as a percentage of phosphorylation in untreated hTau^0N3R^ flies). However, unlike the soluble tau fraction, the hTau in the insoluble (GTO) fraction was largely un-phosphorylated in all animals in which GTOs were produced (Fig. 4b”–f”). Though there is significantly less tau protein in the S3 fractions, the data in Fig. 4a” implies that the lack of phospho-tau immunoreactivity in this fraction, was not due to undetectable total tau levels. This is because, in line with the biochemical analyses presented in Fig. 2, a non-phosphorylation dependent anti-tau antibody detected significant amounts of tau in this S3 fraction (Fig. 4a”). Additionally, equating the total tau levels of the S1, S2 and S3 fractions (by decreasing the amount of tau protein loaded in S1 and S2 by several fold), still gave a positive signal with phospho-tau antibodies in these fractions without any signal in S3 (Supplementary Fig. 9), further implying that the lack of signal in S3 was not due to inadequate tau protein in S3. Thus, the tau proteins contained within the GTOs formed following GSK-3β inhibition are unlikely to be phosphorylated. These findings imply that tau oligomers formed *in vivo* don’t always need to comprise of phosphorylated tau molecules; they can also be made up from non-phosphorylated tau molecules.

### GTO-like structures displayed oligomerisation and lack of β-pleated sheet structure

**Figure 5:**
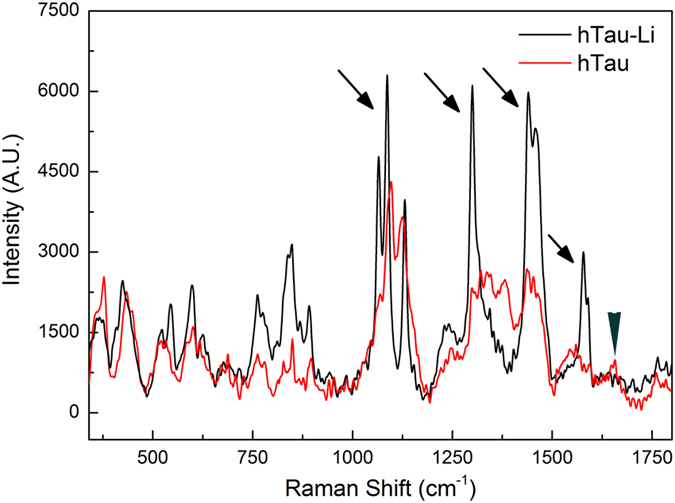
Raman spectroscopy indicates oligomeric structure and lack of β-pleated sheet Raman spectroscopy was carried out on the P2 (detergent-insoluble GTO) fraction prepared from fly heads. The spectrum of Li-treated hTau {*elav*^*C155*^*-Gal4*/Y; UAS- hTau^0N3R^/ + } P2 fraction (black) contained several peaks (arrows) of reduced bandwidth and greater intensity than that from untreated hTau fractions (red), indicating more oligomeric structure in hTau-Li. No peaks are observed at 1665–1690 cm^−1^ (arrowhead) indicating a lack of β-pleated sheet structure or at 980–1000 cm^−1^ indicating lack of phosphorylated species.

To further investigate the chemical nature of these GTO-like structures we carried out Raman spectroscopy. This technique interrogates the vibrations of bonds in molecules generating characteristic spectral profiles. It allows insight into the chemical structure and interactions between different groups and side chains in proteins, providing an analysis of their secondary structure^42^ and aggregated state^43^. We hypothesized that the spectral profile of insoluble fractions generated from hTau flies in which the tau is primarily monomeric, would differ from that of Li-treated hTau flies in which tau oligomers form. Indeed, we found that the insoluble GTO-like structure containing samples (which we know only contain tau and no other proteins – see Supplementary. Fig. 10) provided several spectral markers to confirm their oligomeric nature. For instance specific peaks such as the one at 1382 cm^−1^ that is indicative of disorder^43^ in untreated Tau spectrum disappears while new peaks such as the one at ~540 cm^−1^ characteristic of disulfide linkages appear confirming their formation as one would expect in Tau oligomerisation^44^. The change of state from monomer to oligomeric species is further reinforced by the evolution of the amide II vibration from 1544 cm^−1^ to 1578 cm^−1^. Furthermore, several peaks of reduced bandwidth (i.e. greater sharpness) and greater intensity than those from untreated hTau flies (arrows – Fig. 5) can be observed in the spectra of treated flies. The increased intensity signifies an increase in corresponding bond number, while increased peak sharpness indicates less conformational freedom (more rigidity)^45^ which will occur upon oligomerisation. Such oligomer related peak-changes have been observed by others^43^. Furthermore the Raman spectra of Lithium treated Tau samples shows that not only do the Tau-Li samples have sharper peaks, but that these are also slightly shifted indicating a species with an oligomeric conformation.

All these observations imply that tau in the hTau-Li sample is oligomeric, confirming our biochemical data. Ponceu staining of the P2 fraction, from which the insoluble S3 oligomer fraction is generated for this spectroscopic analysis shows that there is primarily only one band corresponding to the tau protein in that fraction (S3) so the only signal possible is from the tau oligomers (Supplementary. Fig. 10).

Phosphorylation has previously been measured by Raman spectroscopy, and it has been clearly demonstrated that, under basic conditions, a strong peak is evident at around 980 cm^−1^ 46,47. Our Raman spectra recorded on the S3 fraction, which were prepared under basic conditions, do not show the presence of any significant peak at ~980 cm-1 (Fig. 5). The absence of such a peak in our spectra is highly suggestive of the lack of phosphorylation in the tau species that constitute these oligomers.

To probe the secondary structure of our tau oligomers, we studied the spectroscopic traces within regions 1625–1700 cm^−1^ (amide I) and 1220–1300 cm^−1^ (amide III). These are typically used for determining the secondary structure of proteins, although the former is more reliable as it is free from contributions from side-chain vibrations^42^,^45^,^48^. In the Raman spectrum of hTau-Li, there was a near absence of any spectral feature around 1665–1690 cm^−1^ (arrowhead – Fig. 5), indicating the lack of a β-pleated sheet structure. The very weak presence of a peak at ~1650 cm^−1^ (and more so in the untreated hTau spectrum) suggests a helical arrangement of the protein backbone. This is also consistent with the slight increase in intensity of the peak at ~1250 cm^−1^ observed, which indicates an increase in the non-β-sheet structures (such as α-helices or random secondary structures), in the oligomeric protein compared to the monomer^49^. Taken together, these data imply that the GTO-like structures generated following GSK-3β inhibition lack β-pleated sheet structures. Since it is generally presumed that β-pleated sheet structures are toxic, the lack of such structure in our insoluble tau oligomers is consistent with their apparent non-toxicity.

## Discussion

We present here novel data that genetic or pharmacological inhibition of GSK-3β-mediated tau phosphorylation rescues phospho-tau phenotypes, and unexpectedly increases total tau levels and produces insoluble tau oligomers resembling granular tau oligomers (GTO)s. These insoluble tau oligomers are not phosphorylated, and do not contain β-pleated sheet structure. Contrary to prevailing opinion, this demonstrates that oligomeric tau is not necessarily toxic.

### Not all aggregated proteins are toxic

In all proteinopathies there are a variety of soluble and insoluble misfolded proteins. It is now generally accepted that the largest insoluble structures in these diseases (such as neurofibrillary tangles in tauopathies) are not the most toxic species. In the case of tau protein, suspicion has now fallen on small soluble and insoluble oligomers^25^,^26^. However, there is precedent from many aggregate-prone proteins such as beta-amyloid, huntingtin and alpha-synuclein that the smallest insoluble form is relatively protective, while the toxic species is soluble^19^,^20^,^21^. In the case of tau protein this idea is in its infancy, though supportive circumstantial evidence is beginning to emerge. Our data are the first to show that treatments which render tau less soluble rescue tau-induced toxicity, thus demonstrating that small insoluble tau oligomers are associated with neuroprotection *in vivo*. Though our findings do not necessarily imply that tau oligomers are directly neuroprotective, they clearly shows that they are certainly not always toxic when formed *in vivo*.

As we, and others have previously shown, tau phosphorylated at GSK-3β sites is associated with toxicity in both *Drosophila*^9^,^50^ and rodent models^51^. The fact that the insoluble tau oligomers we describe here are not phosphorylated and do not contain β-pleated sheet structure may reconcile the present results with other reports suggesting that tau oligomers are toxic^24^,^30^. We speculate that tau oligomers comprised of non-phosphorylated tau may be non-toxic, whereas those comprised primarily of highly-phosphorylated tau in β-pleated sheet conformation may be toxic^26^.

### Reduction of tau phosphorylation unexpectedly promotes tau aggregation

It is intriguing that reduction of tau phosphorylation in this study promotes tau aggregation, because conventional opinion dictates that hyper-phosphorylation of tau precedes and promotes aggregation. Although one other study is consistent with ours in showing that decreasing GSK-3β activity leads to increased levels of insoluble tau oligomers^33^, many other studies, mostly in rodent models, have conversely found that *increasing* GSK-3β activity promotes tau aggregation^6^,^14^,^52^, and that inhibition of GSK-3β reduces insoluble tau^51^,^53^. Though it is conceivable that our findings, like that of Blard *et al.*^33^, are specific to *Drosophila* models of tauopathy, we do not believe this to be the case. Instead we speculate that the discrepancy between our results and those of others is due to two reasons. Firstly, in studies in which the effect of GSK-3β inhibitors on tau aggregation was analysed^51^,^53^, the insolubility fractionation protocols employed enrich for tau filaments and tangles and actually *loose* small insoluble tau oligomers. As discussed in^26^ insoluble tau oligomers are too small to sediment in the standard high-spin sedimentation spins used in these protocols and yet are too large to enter a SDS-PAGE resolving gel. Hence they would not be detectable unless the insolubility fractionation was specifically chosen to enrich for small insoluble tau oligomers^24^. Thus, though the studies using GSK-3β inhibitors report a reduction of large tau aggregates (such as filaments) it is not possible to conclude whether or not insoluble tau oligomer levels were affected. Perhaps like us, they too may have detected increased insoluble tau oligomer levels following GSK-3β inhibitors if they had used the insolubility fractionation protocols employed for detecting such small insoluble tau species. The second explanation for the discrepancy between our findings and those of others is the increased total amount of tau that we observe after GSK-3β inhibition. Since tau aggregation (at least *in vitro*) is critically dependent upon tau concentration^41^, we suggest that it is the increased tau concentration that drives formation of insoluble tau oligomers in our model. We would argue that increased phospho-tau levels (by inhibiting degradation of phospho-tau) would also lead to tau oligomer formation but in that case these oligomers may be toxic because they would be composed of phosphorylated tau. Indeed this was recently demonstrated in a rodent model of tauopathy^54^.

It will be interesting to investigate the mechanism by which GSK-3β inhibition increases total tau. Like us, others have also shown GSK-3β mediated regulation of tau turnover in both invertebrate and vertebrate models^33^,^55^,^56^,^57^. It is plausible that phosphorylation at some of the GSK-3β sites may be targeting the tau for degradation, and thus under GSK-3β inhibition there is less tau protein turnover, leading to tau accumulation. Other GSK-3β substrates such as β-catenin do indeed signal their own degradation in this manner^58^, and there is precedence for preferential degradation of phosphorylated tau over unphosphorylated tau^55^,^56^.

Though phosphorylation of tau is believed to precede tangle formation, it is intriguing that not all tau proteins derived from paired helical filaments in AD are phosphorylated^59^. Indeed it is speculated that tau proteins found at the core of paired helical filaments are not phosphorylated (either that or phosphorylated epitopes are cleaved) whilst those located at the periphery are^59^. In light of this, it is conceivable that non-phosphorylated tau oligomers, such as the ones we describe in our study, may actually form at an early stage in human brain, and though may not be toxic themselves, they could seed aggregation of more toxic phosphorylated tau species.

### Implications for therapeutic approaches to tauopathy

The failure of amyloid-based therapies in clinical trials has stimulated research into tau-based targets for the treatment of tauopathies like Alzheimer’s disease. Two major tau-based strategies are directed at inhibiting GSK-3β-mediated tau phosphorylation, and reducing tau aggregation/oligomerisation by various means^34^,^60^. Examples of the latter include chemicals such as rhodanines, N-phenylamines, phenylthiazolhydrazides, and methylene blue that can inhibit or dissolve tau aggregates^61^. Alternatively, tau might be cleared by tau vaccination approaches^62^.

Our finding that inhibition of tau phosphorylation leads to production of insoluble tau oligomers raises a cautionary note regarding the therapeutic strategies aimed at reducing tau phosphorylation. Our results suggest the outcome of this strategy may conflict with the aim of the other key therapy if, as we show, inhibition of tau phosphorylation leads to formation of tau oligomers.

Additionally, our finding that tau oligomers are not always toxic has implications for the effectiveness of strategies aimed at reducing tau aggregation. Such strategies would be ineffective if the tau oligomers that they are clearing are not toxic. Furthermore, these approaches might even be counter-productive if solubilising aggregated tau releases the more toxic species. Our results underscore the importance of establishing which species of oligomeric tau are toxic, neutral or protective to determine the most appropriate target for effective tau-based therapies.

## Conclusion

The mechanism(s) by which abnormal tau causes toxicity in tauopathies is not clear. Although it is established that abnormal tau phosphorylation plays a critical role, the role of tau aggregation, and in particular oligomerisation, is less well understood. We have found that reduction of tau phosphorylation promotes tau aggregation, and that this is, surprisingly, associated with rescue of neuronal dysfunction *in vivo*. This has profound implications for therapeutic approaches aimed both at inhibiting tau phosphorylation and reducing tau aggregation.

## Materials and Methods

### Flies

*Drosophila melanogaster* expressing the GAL4 drivers *elav*^*C155*^*-Gal4* or *D42-Gal4* were crossed with either wild-type Oregon-R flies (wt) as a control, or with flies transgenic for human 3-repeat tau (*UAS-hTau*^*0N3R*^from Bloomington Stock centre), with or without UAS-sgg^*^ (*UAS-sggS9A* from Bloomington Stock centre) or *UAS-sgg*^*DN*^(Dr. D Allan UBC, Canada). Flies were raised at 25 °C on standard fly food with or without 20 mM LiCl or 20 μm AR-A01448 (Sigma-Aldrich). For all experiments, equivalent numbers of males and females were utilized for each condition compared.

### Solubility fractionation of granular tau oligomers

10 fly heads (0-3d flies) were pooled and homogenized in 40 μl aqueous buffer (50 mM Tris-HCl pH 7.4, 175 mM NaCl, 1 M sucrose, 5 mM EDTA) at 4 °C, and centrifuged at 1,000 g for 2 minutes to remove unhomogenized debris. The supernatant was then centrifuged for 2 h at 200,000 g (4 °C). The resulting supernatant (S1) is the aqueous-soluble fraction. Pellet (P1) was resuspended in SDS buffer (50 mM Tris-HCl pH 7.4, 175 mM NaCl, 5% SDS) and centrifuged for 2 h at 200,000 g (25 °C). Supernatant (S2) is the detergent-soluble fraction. P2 was washed by repeating this spin and, after discarding the supernatant, P2 was resuspended in buffer containing 8 M urea (50 mM Tris-HCl pH 7.4, 175 mM NaCl, 8% SDS, 8 M urea) with agitation for 18 h at room temperature. S3 is the detergent-insoluble fraction.

### Western blotting

Samples of pooled fly heads (0-3d flies) were either prepared for solubility fractionation, as above, or homogenized in buffer containing protease, kinase and phosphatase inhibitors (150 mM NaCl, 50 mM MES, 1% triton-X 100, 1% SDS, 2 μg/ml leupeptin, 2 μg/ml aprotinin, 100 μg/ml PMSF, 30 mM NaF, 40 mM 2-glycerophosphate, 20 mM sodium pyrophosphate, 3.5 mM sodium orthovanadate, 10 μM staurosporine). Samples were heated for 5 minutes at 95 °C in Laemmli buffer, separated by 10% PAGE, and transferred to PVDF membrane (Amersham). Blots were probed with the following primary antibodies: anti-human-tau antibody (Dako, 1:15,000), anti-human-tau N-terminal (Abcam, 1:1000), tau-1 (Millipore, 1:2,000), PHF-1 (Peter Davies, 1:500), AT8 (Source Biosciences, 1:800), AT180 (Source Biosciences, 1:100), MC1 (Peter Davies, 1:200), or anti-pS262 (Invitrogen, 1:1,000), followed by HRP-conjugated anti-rabbit secondary antibody (Cell signalling) and Chemiluminescent substrate (Amersham). Band densities were measured using Image J.

### Transmission Electron Microscopy, *in situ*

Filleted L3 larvae were fixed (3% glutaraldehyde, 4% formaldehyde in 0.1 M PIPES buffer, pH 7.2) for 1 hour. Specimens were rinsed in 0.1 M PIPES buffer, post-fixed in 1% buffered osmium tetroxide (1 hour), block-stained in 2% aqueous uranyl acetate (20 min), dehydrated in ethanol and embedded in Spurr resin (EM Science). Ultra-thin sections were cut through the nerves at the base of the ventral-cord on a Leica OMU 3 ultramicrotome. The sections were stained with Reynolds lead stain and viewed on a Hitachi H7000 transmission electron microscope with SIS Megaview-III digital camera. The number of microtubule profiles per axon was counted in cross sections of peripheral nerves. Comparable regions of peripheral nerves were studied and all animals were processed in parallel. All visible axons were analysed in 5 animals per condition (approximately 240 axons per animal), with the experimenter blinded to condition.

### Transmission Electron Microscopy with *in situ* immuno-gold labeling

Filleted CNS preparations from L3 larvae were fixed in 4% PFA (15 min), then the anterior half of the fillet was cut away and post-fixed overnight in fresh 4% PFA. Samples were cryoprotected by incubation in a glycerol series up to 30%. Samples were briefly plunge-frozen in liquid ethane (−170 °C). Frozen samples were incubated in 1.5% uranyl acetate in methanol at-90 °C for 30 h, then at −45 °C for11 h. This was followed by infiltration with a series of resin:methanol mixtures at −45 °C, culminating in pure Lowicryl HM-20 resin (EM Science) overnight. Specimens were polymerized by UV light (24 h, −45 °C), then the temperature increased to 0 °C over 9 h. Gold/silver sections were cut through the proximal peripheral nerves on a Leica ultramicrotome, and transferred to formvar-coated Ni grids (EM Science). Immunochemistry was carried out as described^28^ using anti-tau (Sigma, polyclonal T6402 1:100), and secondary goat-anti-rabbit 10 nm gold (EM Sciences). The specimens were viewed on a FEI Tecnai G2 Spirit Transmission Electron Microscope.

### Transmission Electron Microscopy with immuno-gold labelling of fractionated protein

The insoluble protein fraction (P2 from the solubility fractionation method) was resuspended in 20 μl distilled water, and 5 μl spotted onto carbon-formvar copper grids (EM Science) for 2 min. Grids were blocked in 1% milk in PBS; incubated in anti-human-tau (Sigma) or anti-v-glut at 1:200 in 1% milk in PBS (60 min room temperature); washed in PBS; incubated in gold-conjugated anti-rabbit secondary antibody (EM Sciences - 1:30) in 1% milk in PBS (60 min room temperature); washed in PBS then water; negative stained with 2% uranyl acetate (10 s). Grids were viewed as above.

### Atomic force microscopy

20 brains of L3 larvae per sample were dissected on ice and homogenized in 500 μl buffer (50 mM MES, 150 mM NaCl, 1% triton-X, protease inhibitor cocktail), centrifuged (2 min 2,000 g) and unhomogenized material discarded. Samples were immuno-precipitated as follows: preclear in 20 μl protein A/G beads (Sigma) at 4 °C 1 hour; incubate with 1 μl anti human-tau (Dako) at 4 °C 1 hour; add 60 μl beads and incubate at 4 °C 1 hour; wash beads 3 times in buffer; reduce precipitated sample in 20 μl Laemlli buffer for 5 min 90 °C. Precipitates were spotted onto freshly cleaved mica discs (Agar Scientific), incubated for 2 minutes, rinsed with 200 μl ultrapure water and dried with compressed air. Samples were imaged in air with a digital multimode Nanoscope III AFM (www.veeco.com) operating in tapping mode with an uncoated silicon cantilevers (FM-W, Nanoworld Innovative Technologies, Switzerland, nominal spring constant 2.8 N/m) with set points of 0.6–0.8 at a scan frequency of 3 Hz. Oligomer sizes were determined by cross-sectional height analysis of individual aggregates.

### Raman Spectroscopy

Insoluble protein fractions (P2 from solubility fractionation protocol above), from hTau and hTau-Li brains were placed on slides. An InVia Renishaw Raman microscope system was used for obtaining spectra. A 633 nm laser was used for excitation with an acquisition consisting of 3 exposures of 10 s each. Spectra were processed with Wire 3.1 software: fluorescence background was subtracted and spectra were smoothed.

### Locomotor contractions assay

Htau^0N3R^ expression was driven by the motor neuron specific drive *D42-GAL4*. Third instar L3 larvae were allowed to crawl freely on an agarose plate under standardized lighting conditions. Their crawling behavior was recorded using standard video recording equipment and body wall contractions undertaken in one min were counted.

## Additional Information

**How to cite this article**: Cowan, C. M. *et al.* Rescue from tau-induced neuronal dysfunction produces insoluble tau oligomers. *Sci. Rep.* **5**, 17191; doi: 10.1038/srep17191 (2015).

## Supplementary Material

Supplementary Information
