# Supplementary material for: Rescue from tau-induced neuronal dysfunction produces insoluble tau oligomers
Source: Sci Rep. 2015 Nov 26;5:17191. doi: 10.1038/srep17191 (PMC4660438; doi:10.1038/srep17191)
Supplement: Supplementary Information [file srep17191-s1.pdf]

Rescue from tau-induced neuronal dysfunction produces insoluble tau oligomers

Catherine M. Cowan, PhD[1], Shmma Quraishe, PhD[1], Sarah Hands, PhD[1], Megan Sealey[1], Sumeet Mahajan, PhD[2], Douglas W. Allan, PhD[3], Amritpal Mudher, DPhil[1*].

Cowan et al Supplementary Figure 1

Quantification of GTO-like structures in EM sections

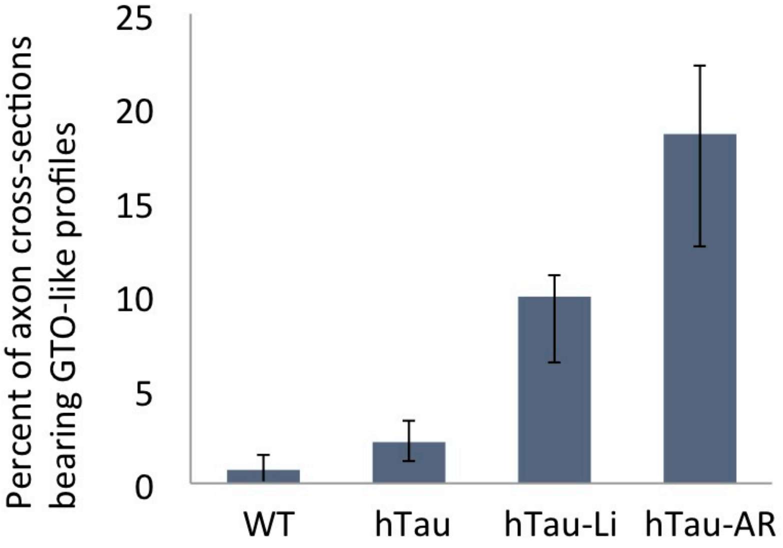

$p < 0.01$, 1-way ANOVA

n = >150 axon profiles from >12 fields of view from 3 animals per condition

Supplementary Fig. 1: Quantification of GTO-like structures following GSK-3β inhibition Percentage of axon cross sections in L3 peripheral nerve bundles bearing GTO-like structures in wt or $hTau^{0N3R}$ {$elav^{C155}$-*Gal4*/Y; UAS- $hTau^{0N3R}$/+} larvae after treatment with LiCl or AR-A01448 compound. Though there were a small percentage of axons bearing GTO-like structures in some $hTau^{0N3R}$ expressing larvae, these were significantly more prevalent following inhibition of GSK-3β. More than 150 axon profiles were examined in more than 12 fields of view from 3 animals per condition. $P<0.01$; 1-way ANOVA.

Cowan et al Supplementary Figure 2

Inhibition with LiCl or AR-A01448 protects against tau-induced defects in locomotion

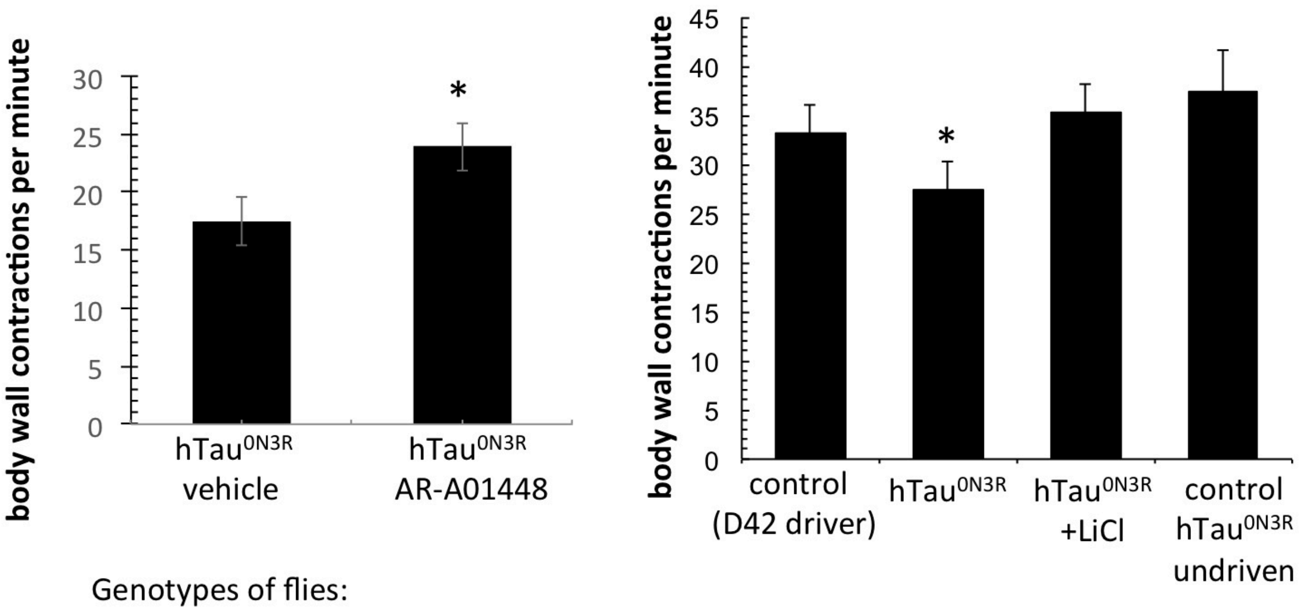

Genotypes of flies:

control (D42) = w ; D42-Gal4/+

D42 hTau = w ; D42-Gal4/+ ; UAS-hTau^{0N3R}/+

control (hTau) = w ; ; UAS-hTau^{0N3R}/+

n = 14-20

* = 0.04 for AR cpd and 0.05 for LiCl (students t test)

Supplementary Fig. 2: Inhibition of GSK-3β with LiCl or AR-A01448 protects against tau-induced defects in locomotion. Body wall contractions were significantly increased by treatment of third instar hTau^{0N3R} –expressing {*D42-Gal4*/+; UAS- hTau^{0N3R}/+} larvae with either AR-A01448 compound (a) or LiCl (b). This confirms our previous findings that GSK-3β inhibition rescues tau-mediated locomotor phenotypes. n=14-20; $p < 0.05$ students t-test.

Cowan et al Supplementary Figure 3: Treatment with AR compound restores cytoskeletal integrity

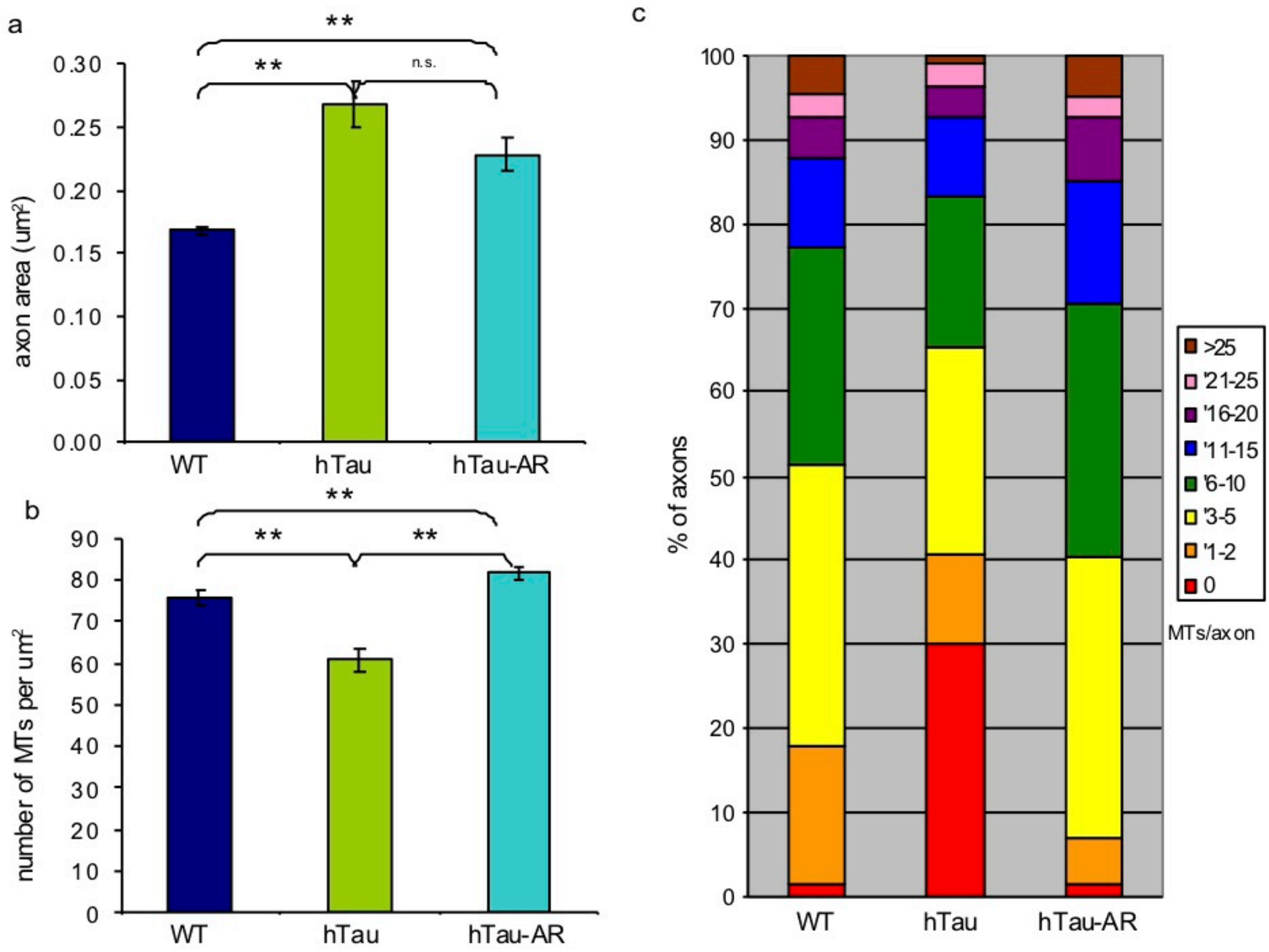

<u>Supplementary Fig. 3: Inhibition of GSK-3β with AR-A01448 restores cytoskeletal integrity.</u> Further analysis of electron micrographs of Drosophila peripheral nerves. a) The average axon diameter in hTau^{0N3R} flies {*elav*^{C155}-*Gal4*/Y; UAS- hTau^{0N3R}/+} was greater than controls, and there was a trend towards the rescue of this phenotype upon AR-A01448 treatment. b) The number of microtubules per unit area of axon was significantly decreased in hTau^{0N3R}-expressing animals compared to control, and this was rescued by AR-A01448 treatment. c) The data in Fig 1m (number of microtubules per axon) expressed as the percentage of axons exhibiting various ranges of the numbers of intact microtubule profiles. Note that 30% of hTau^{0N3R}-expressing axons contain no visible microtubule profiles, while this is rare in WT or hTau^{0N3R} - AR-A01448 axons.

Cowan et al Supplementary Figure 4
Treatment with LiCl or AR-A01448 does not increase levels of *Drosophila* tau or tubulin

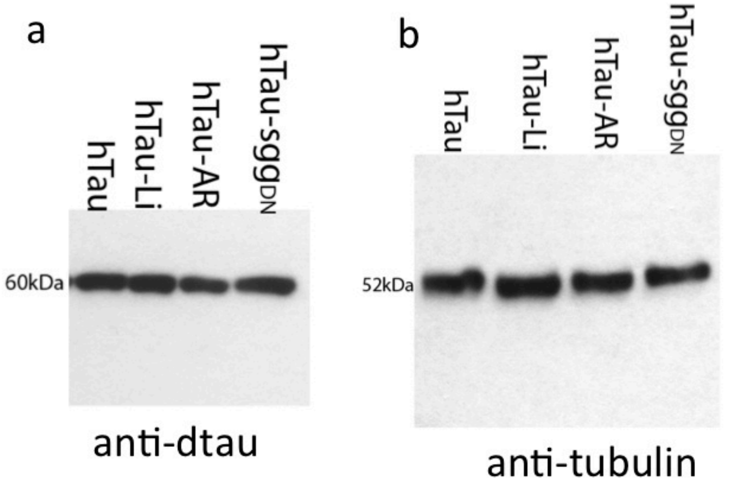

Supplementary Fig. 4: Inhibition of GSK-3β does not non-specifically increase levels of endogenous proteins *Drosophila* tau or tubulin. Loading controls for blots shown in Fig 1o-n showing that following either pharmacological or genetic inhibition of GSK3β, there was no non-specific increase in protein expression as seen by probing total lysates for *Drosophila* tau (a) and b-tubulin (b). This shows that the increase seen in $hTau^{0N3R}$ levels (Fig 1 o-q) following GSK3β inhibition is specific to hTau.

Cowan et al Supplementary Figure 5
LiCl treatment does not increase the amount of GFP in Elav-Gal4 ; UAS-GFP flies (n= 4 blots)

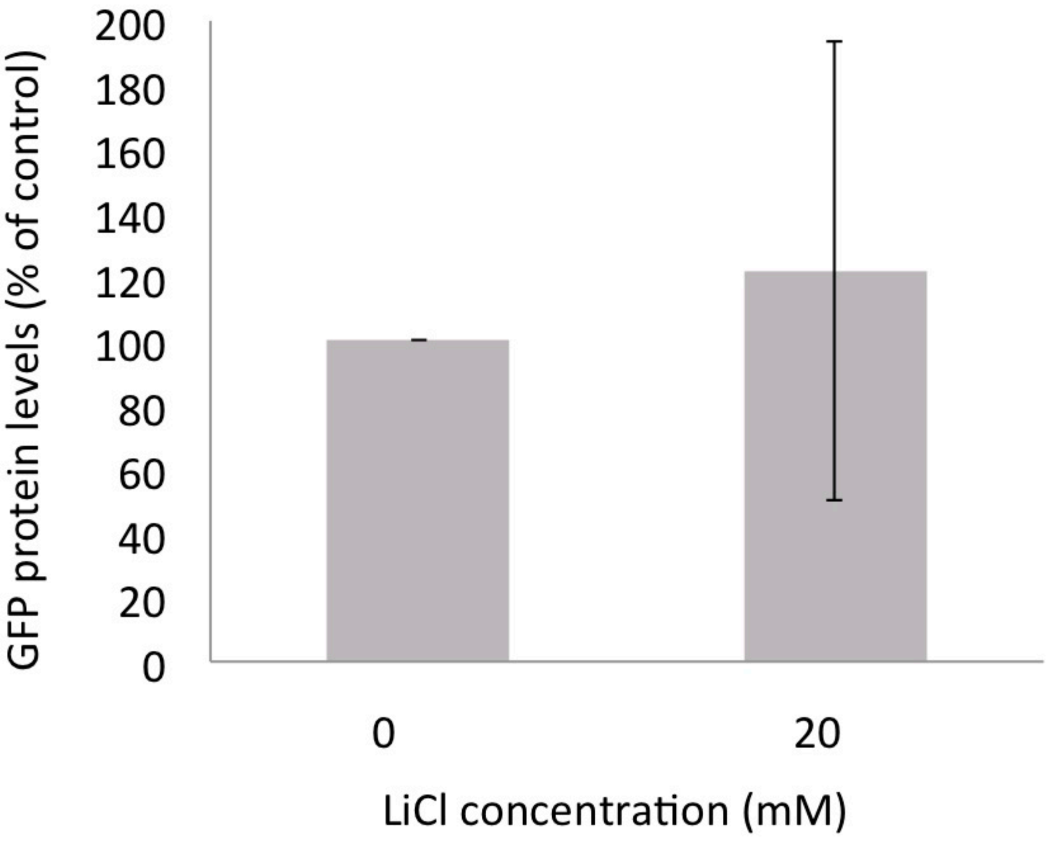

Supplementary Fig. 5: LiCl treatment does not non-specifically increase levels of transgenes. Treatment of transgenic lines expressing GFP {*elav*^{C155}-*Gal4*/Y; UAS-GFP/+} with LiCl does not lead to an increase in expression of GFP. This shows that the increase in hTau^{0N3R} brought about by pharmacological or genetic inhibition of GSK-3β is not due to a non-specific effect of these drugs on the UAS-GAL4 expression system. (n=4 blots).

Cowan et al Supplementary Figure 6

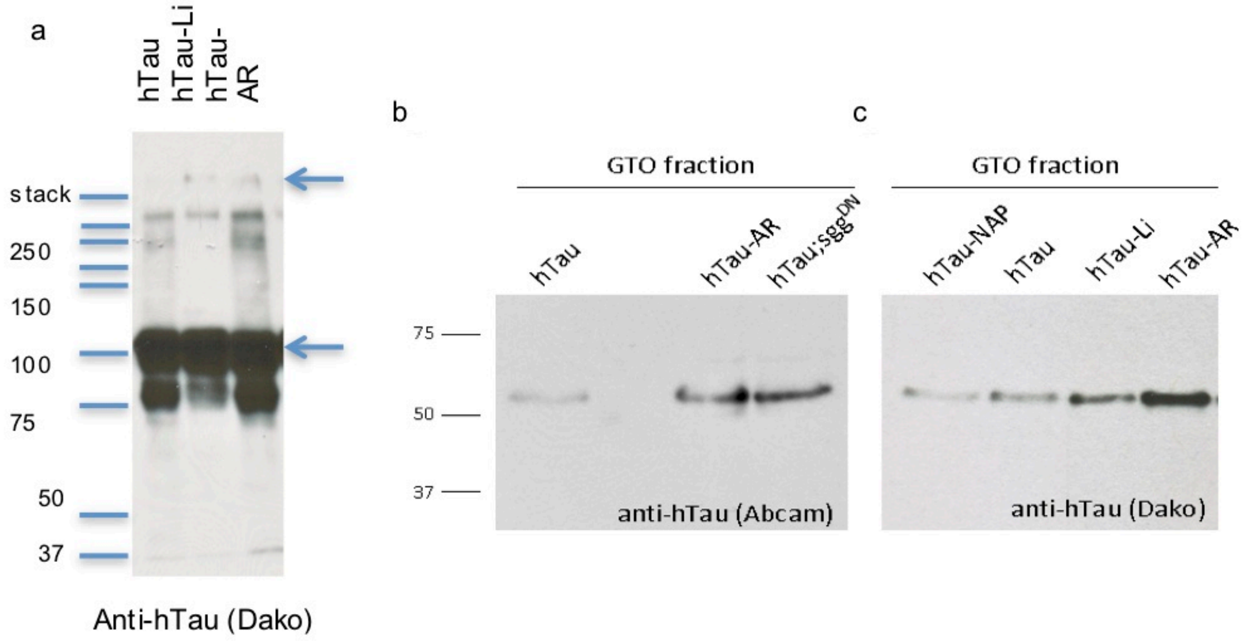

<u>Supplementary Fig. 6: Insoluble tau species evident in S3 fraction</u>. Insoluble material in the stacking gel (not entering the resolving gel) was detected in fly lysates with anti-tau antibody after treatments of htau^{0N3R} flies {*elav*^{C155}-*Gal4*/Y; UAS- GFP/+} which caused electron-dense structures (LiCl and AR-A01448 compund) but not before. Upper arrow: insoluble material in the stacking gel. Lower arrow: overexposed monomer. b) Western blot of fly lysates and insoluble GTO fractions generated from these lysates probed with a monoclonal antibody against the N-terminal portion of human tau (Abcam), indicating that the insoluble tau was also recognized by another tau antibody. c) Not all treatments which rescue the tau-induced phenotype also produce GTOs, as evidenced by western blot of the insoluble fraction from hTau flies treated with 2.5 ug/ml NAP. Like LiCl and the AR-A01448 compound, NAP rescues the tau phenotype in our model[38], yet, unlike the GSK-3β inhibitors, it does not cause the formation of insoluble tau.

Cowan et al Supplementary Figure 7
Positive and negative controls for solubility assay

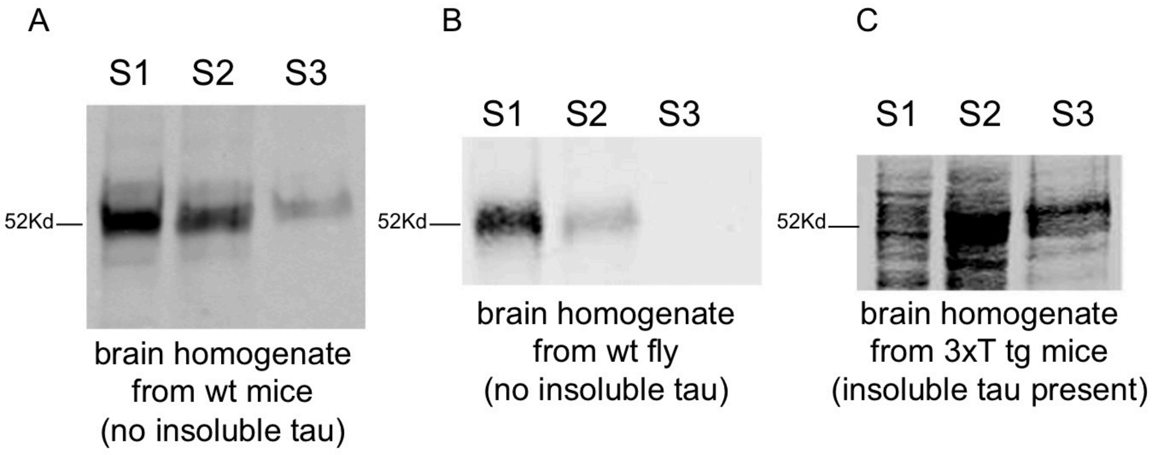

Supplementary Fig. 7: Positive and negative controls for insolubility assay. Brain homogenate from wt mice (a), wt flies (b) and triple transgenic tau mice (c) were fractionated with our insolubility protocol. The lack of significant signal in the S3 fraction from homogenates of wt mice and wt flies is in line with the fact that most tau in these animals is soluble, with only a small amount of insoluble tau evident. In contrast, the strong signal in the S3 fraction obtained from homogenates of triple transgenic tau mice (3xT tg) demonstrates what is widely known, that there is a considerable amount of insoluble tau in the brains of these animals. These blots serve as the positive and negative controls for the solubility protocol used to generate the data in Figures 2 and 4.

Cowan et al Supplementary Figure 8
negative controls for immuno-EM

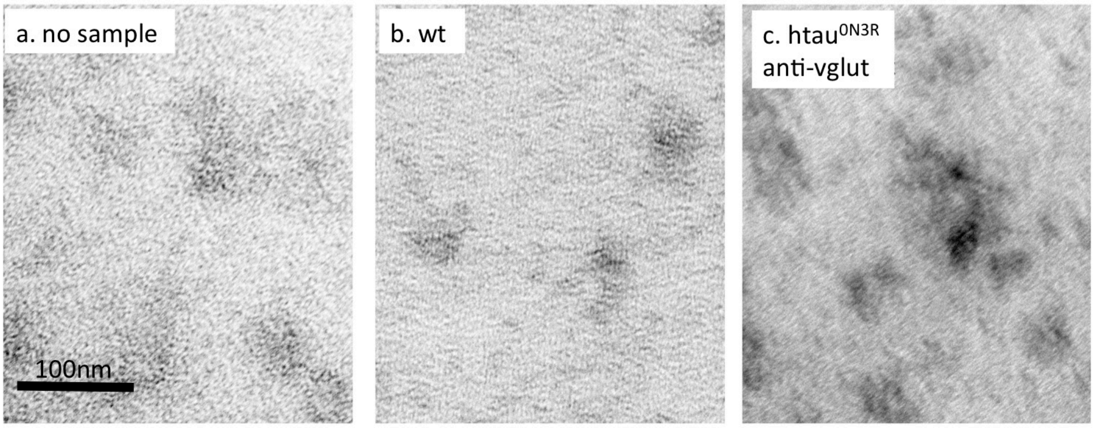

Supplementary Fig. 8: Negative controls for immuno-EM. The absence of signal in insoluble pellets generated from brains of LiCl treated hau^{0N3R} (a and c) or wt (b) control animals {*elav*^{C155}-*Gal4*/Y; UAS- hTau^{0N3R}/+}. In (a) no antibody was used; in (b) an anti-tau antibody was used but not much signal is evident in wt controls. In (c) the pellets from LiCl treated hTau^{0N3R} animals were probed with a non-specific antibody – anti-vglut, again showing very little immunoreactivity. These controls show that the presence of immuno-EM signal in Figure 3 a-c was not due to a non-specific primary antibody effect.

Cowan et al Supplementary Figure 9

lack of phospho-tau signal in S3 is not due to insufficient protein in S3

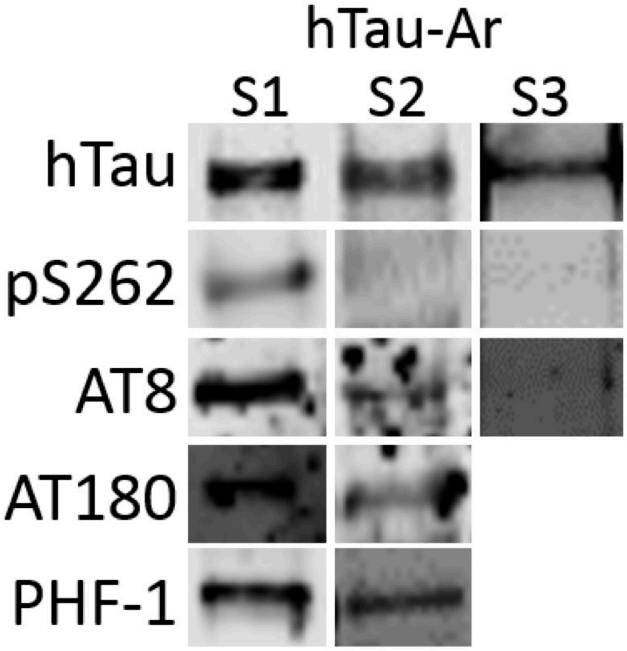

Supplementary Fig. 9: lack of phospho-tau signal in S3 fractions is unlikely to be due to inadequate tau levels in S3. Equating the tau levels in S1, S2 and S3 fractions from hTau^{0N3R} {*elav*^{C155}-*Gal4*/Y; UAS- hTau^{0N3R}/+} animals treated with AR-A01448 by reducing volumes of S1 loading 60 fold, reducing volumes of S2 loading 6 fold and increasing S3 loading 10 fold. This gives comparable level of total tau in each fraction, as probed by an htau polyclonal antibody (Dako polyclonal). This amount of tau is sufficient to give a positive signal with a variety of phospho-tau antibodies in the S1 and S2 fractions, but is still not picked up by any phosho-tau antibody in the S3 fraction. This implies that the lack of signal in the S3 fractions of AR-A01448 compound treated hTau^{0N3R} brains is not due to undetectable total tau in that fraction. It is likely to be due to inadequate phosphorylation of that tau in these S3 fractions.

Supplementary Fig. 10 Ponceu staining of soluble tau (S1) and aggregated tau/GTO fraction (S3). Whereas there are quite a few proteins present in the soluble S1 fraction, there is only a faint band in the hTau {*elav*^{C155}-*Gal4*/Y; UAS- hTau^{0N3R}/+} LiCl and AR-A01448 compound treated lanes in the S3 fraction (arrow). When this fraction is probed with an anti-hTau antibody, then only hTau immunoreactivity is evident (Fig 2a and Fig 3a) collectively showing that hTau is the only protein found in this fraction following our aggregated tau/GTO fractionation. This is the fraction on which Raman spectroscopy was carried out so it is highly likely that all the spectra thus obtained related only to tau protein found in this fraction.

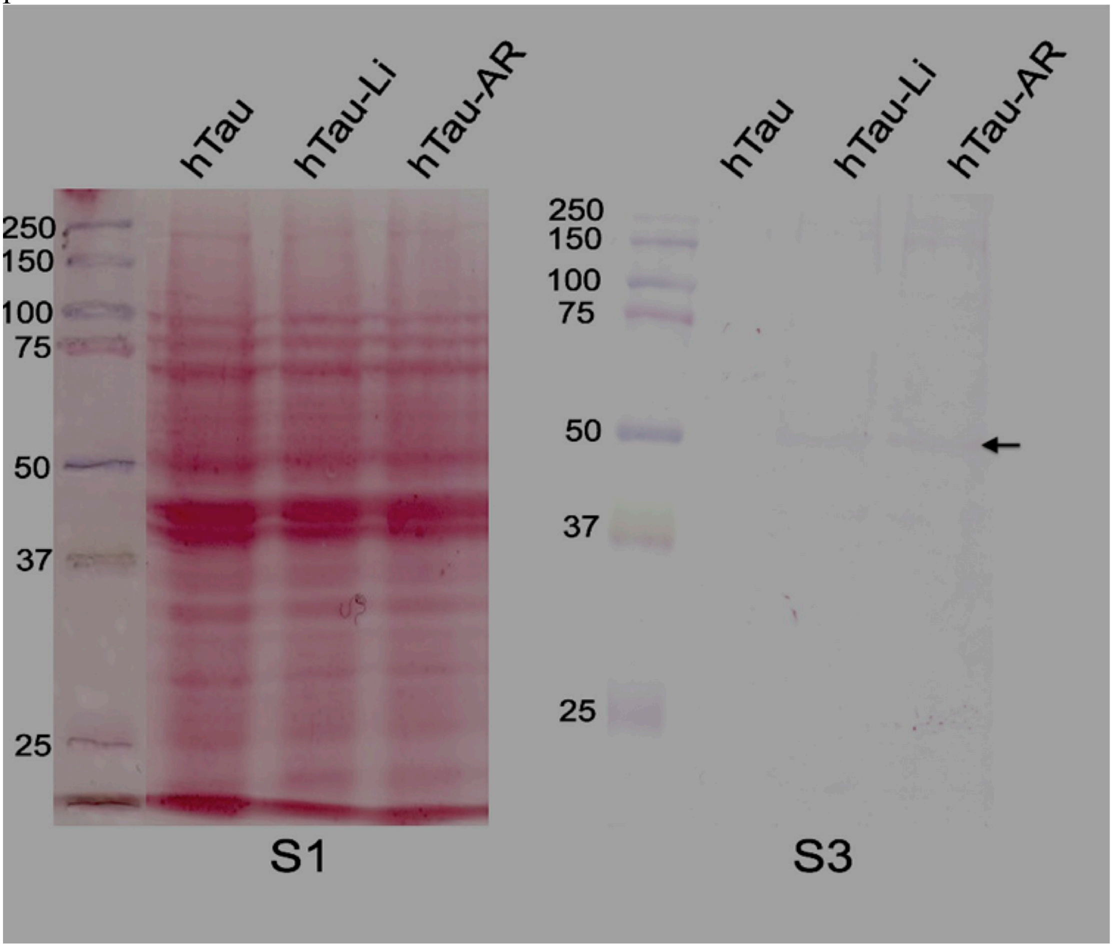

Supplementary Figure 10
